# Deproteinization-Induced Deterioration of the Mechanical and Tribological Behaviors of Mature Giant Panda Enamel

**DOI:** 10.3390/ani16142270

**Published:** 2026-07-22

**Authors:** Zheng Fang, Haojie Xu, Yifan Wen, Yipeng Jin

**Affiliations:** College of Veterinary Medicine, China Agricultural University, Beijing 100193, China; fangzcau@foxmail.com (Z.F.);

**Keywords:** giant panda, enamel, deproteinization, wear resistance

## Abstract

The giant panda (*Ailuropoda melanoleuca*) relies on bamboo as its principal food source, exposing its enamel to sustained indentation, shearing, and abrasive loading. Mature enamel is highly mineralized but retains a small residual organic phase, whose contribution to wear resistance remains insufficiently defined. A gradient deproteinization model of mature giant panda enamel was established using KOH treatment. Thermogravimetric analysis (TGA), Fourier-transform infrared spectroscopy (FTIR), X-ray photoelectron spectroscopy (XPS), and scanning electron microscopy (SEM) were used to verify organic-phase removal and mineral-framework preservation. Nanoindentation, indentation creep, nanoscratch testing, atomic force microscopy (AFM), and SEM were then used to evaluate mechanical response, scratch resistance, and surface morphological evolution. The mass loss within 200–450 °C decreased with increasing KOH exposure and reached a plateau after 9 days. FTIR showed reduced organic-related or surface-sensitive bands, whereas phosphate bands were retained. XPS revealed decreased C and N contents; increased O, Ca, and P contents; and a higher Ca/P ratio. Deproteinization decreased the elastic modulus, shifted load–displacement curves toward greater indentation depth, and reduced the relative creep index. The critical load in nanoscratch testing decreased from 12.53 ± 0.35 mN to 6.07 ± 0.77 mN, while scratch depth, width, and residual depth increased. AFM showed a rightward shift in aggregate-size distribution and increased roughness. SEM revealed a transition from shallow plowing grooves to complex damage involving cracks, debris accumulation, and local spallation. Therefore, KOH treatment for 9 days provided an effective deproteinization endpoint for mature giant panda enamel. These findings suggest that the residual organic phase may contribute to surface interfacial continuity, local deformation accommodation, scratch-damage resistance, and reduced permanent damage accumulation. This material role may contribute to the adaptation of giant panda enamel to the mechanical demands of a bamboo-based, high-wear diet.

## Simple Summary

Giant pandas eat mainly bamboo, a fibrous and abrasive food that repeatedly loads and wears their teeth. Tooth enamel is mostly mineral but retains a very small organic component. The role of this material in helping giant panda enamel resist damage is not fully understood. In this study, we gradually removed organic material from mature giant panda enamel and measured changes in stiffness, gradual deformation under a constant load, scratch resistance, and surface damage. Removing the organic component reduced enamel stiffness and its ability to accommodate deformation over time. It also lowered the load required to initiate scratch damage and produced wider and deeper scratches, greater surface roughness, cracking, and local material loss. These findings suggest that the small residual organic component helps maintain connections between mineral structures and limits permanent surface damage. This contribution may help giant panda teeth withstand repeated wear during bamboo processing. The results improve understanding of how biological materials combine mineral and organic components to achieve durability and may inform future studies of animal dental health and wear-resistant materials.

## 1. Introduction

Dental enamel is the outermost mineralized tissue of the mammalian crown and provides the primary barrier against contact loading, abrasion, and fracture during mastication [1,2]. Mature enamel is composed predominantly of hydroxyapatite crystallites, with minor amounts of organic components and water [3]. Its mechanical behavior is not determined solely by mineral content, but is also influenced by crystallite orientation, prism and interprismatic architecture, hierarchical decussation, hydration state, and intercrystalline organic matter [4]. Under repeated contact loading and tangential friction, enamel must resist indentation deformation, crack initiation, abrasive cutting, and surface spallation [5]. Therefore, hardness or elastic modulus alone cannot fully describe its long-term service performance.

The giant panda (*Ailuropoda melanoleuca*) has an unusual feeding ecology among ursids, as it consumes bamboo as its dominant food source. Bamboo processing requires repeated biting, shearing, and grinding, thereby imposing high-frequency mechanical loading and abrasive wear on the enamel surface [6,7]. Previous studies have shown that giant panda enamel has substantial load-bearing capacity, damage tolerance, hydration-associated recovery and wear resistance [8]. These properties may be related to its enamel-prism arrangement, hierarchical architecture, and hydration-mediated interfacial response. However, whether the small amount of residual organic matter in mature giant panda enamel contributes directly to surface interfacial stability and wear resistance remains unclear.

Mature enamel has long been regarded as a nearly inorganic tissue. Nevertheless, residual organic components may persist in intercrystalline spaces, prism sheaths, surface interfaces, and the dentin–enamel junction region [9,10]. Proteomic and matrix-layer analyses further indicate that enamel-derived proteins or peptides can remain detectable in erupted or mature enamel [11,12]. Experimental studies on enamel organic components indicate that proteins influence crack-growth resistance and surface protection [13,14]. Deproteinization studies further show that removal or alteration of the organic matrix can affect enamel structure, mechanics and erosion response [15,16]. These findings suggest that the residual organic phase is not merely a passive developmental remnant, but may function as a low-abundance component involved in mature-enamel microstructural stability and damage regulation.

Most available deproteinization studies have focused on human or bovine enamel and have commonly used a single endpoint or short-term surface pretreatment [15,17]. Comparative chemical studies also show that enamel composition differs among common mammalian models [18]. Giant panda enamel represents a distinct biological hard tissue formed under a unique combination of high-wear bamboo feeding and ursid phylogenetic background [6,8]. Establishing a gradient deproteinization model for mature giant panda enamel and correlating the degree of organic-phase removal with mechanical properties, scratch damage, and nanoscale surface morphology may clarify the contribution of the residual organic phase to the wear-resistant phenotype of this enamel [7].

In this study, a KOH-based gradient deproteinization model was established and validated using TGA, FTIR, XPS, and SEM. Nanoindentation, indentation creep, nanoscratch testing, AFM, and SEM were subsequently used to characterize the mechanical and tribological changes induced by deproteinization.

## 2. Materials and Methods

### 2.1. Enamel Sample Collection and Preparation

Six molars from three naturally deceased adult giant pandas were used in this study. The animals included two females and one male, aged 4–8 years. Molars were eligible when the occlusal enamel was grossly intact and free of visible carious lesions. Teeth with severe cracks, large enamel defects, or extensive structural loss were excluded. Surface soft-tissue residues were removed, and the samples were ultrasonically cleaned at 80 Hz and 25 °C for 20 min. The cleaned teeth were alternately rinsed with distilled water and absolute ethanol and then stored in Hank’s balanced salt solution (Beijing Solarbio Science & Technology Co., Ltd., Beijing, China) at 4 °C for no longer than 30 days before preparation.

The roots and dentin were removed under water cooling, and enamel blocks containing the occlusal enamel surface were prepared. Each block was approximately 2 mm × 2 mm × 2 mm. The enamel blocks were embedded in self-curing denture base resin with the enamel test surface positioned horizontally upward. The exposed surface was sequentially ground using P400, P800, P1500, P2000, and P3000 waterproof abrasive papers and polished with W7, W3.5, W2.5, and W0.5 diamond polishing pastes to obtain a mirror-like surface for subsequent characterization.

### 2.2. Gradient Deproteinization Treatment

The prepared enamel samples were immersed in 0.004 mol/L KOH solution (Shanghai Macklin Biochemical Co., Ltd., Shanghai, China) (pH = 11) for gradient deproteinization. The treatment durations were 0, 3, 6, 9, and 12 days. The 0-day group served as the untreated control. During treatment, samples were fully immersed in the solution. At each predetermined time point, specimens were removed, thoroughly rinsed with distilled water, transferred immediately to fresh Hank’s balanced salt solution, and stored fully immersed at 4 °C until testing. Specimens were removed from solution only immediately before mechanical or morphological measurement to limit drying-induced cracking.

### 2.3. TGA, FTIR, XPS, and SEM Validation of the Deproteinization Model

Thermogravimetric analysis was performed using a TGA 550 instrument (TA Instruments, New Castle, DE, USA). Three separately prepared samples were analyzed per group (*n* = 3), and each sample weighed at least 20 mg. After drying, the samples were placed in crucibles and heated from 30 °C to 750 °C at 5 °C/min in air. TG and derivative thermogravimetric curves were recorded, and the mass loss within 200–450 °C was calculated. This temperature interval was used to represent a composite mass-loss signal dominated by thermal decomposition of residual organic components.

FTIR spectroscopy was performed on three representative enamel blocks from each of the 0-day and 9-day groups (*n* = 3 per group) using a Nicolet iS20 spectrometer (Thermo Fisher Scientific, Waltham, MA, USA) in attenuated total reflectance mode. Spectra were acquired over 400–4000 cm^−1^ with a resolution of 4 cm^−1^. Changes in organic-related absorption bands and phosphate/carbonate mineral bands were used to assess surface chemical characteristics after deproteinization.

XPS was performed using a K-Alpha spectrometer (Thermo Fisher Scientific, Waltham, MA, USA) with an Al Kα X-ray source and a spot size of 400 μm. Three representative enamel blocks from each of the 0-day and 9-day groups were analyzed (*n* = 3 per group). Survey spectra were acquired from the 0-day and 9-day groups, and the atomic percentages of C, N, O, Ca, and P were quantified. SEM observations were performed using a MIRA LMS scanning electron microscope (TESCAN, Brno, Czech Republic). For SEM, three representative blocks per group were gold sputter-coated and examined.

### 2.4. Nanoindentation and Indentation Creep Testing

Nanoindentation was performed using a G200 nanoindenter (KLA Corporation, Milpitas, CA, USA). Four separately prepared enamel blocks were analyzed per group (*n* = 4 block-level replicates). All tests were conducted under dry conditions at room temperature, and the continuous testing time for a single sample was controlled within 30 min. For standard indentation, the maximum load was 10 mN, the holding time at peak load was 2 s, and the loading rate was 0.5 mN/s. Five spatially separated indents were made on each block as technical replicates, and their mean was used as the block-level value. Load–displacement curves were recorded, and indentation elastic modulus and hardness were calculated. Nanoindentation has been widely used to evaluate local modulus, hardness, and microscale mechanical response in dental enamel.

Indentation creep testing was performed with a maximum load of 10 mN. Four separately prepared enamel blocks were analyzed per group (*n* = 4 block-level replicates). The load was applied over 60 s and held at 10 mN for 60 s, followed by unloading to 0.5 mN and holding for 100 s for thermal-drift correction. The indentation depth at the beginning of the holding period was defined as h_1_, and that at the end of the holding period was defined as h_2_. Creep displacement was calculated as Δh = h_2_ − h_1_, and the indentation creep index was calculated as CIT = [(h_2_ − h_1_)/h_1_] × 100%. Three spatially separated creep tests were performed per block as technical replicates, and their mean was used for block-level analysis. This protocol was used to characterize time-dependent deformation of enamel under constant load.

### 2.5. Nanoscratch Testing

Nanoscratch testing was performed using the scratch mode of the G200 nanoindenter to evaluate surface tribological behavior and scratch resistance. Four separately prepared enamel blocks were analyzed per group (*n* = 4 block-level replicates). The maximum normal load was 100 mN, the scratch length was 200 μm, and the displacement rate was 400 μm/min. Frictional response and depth signals were continuously recorded to obtain surface roughness, critical load, depth at critical load, scratch width, and residual scratch depth. Three spatially separated scratches were made per block as technical replicates, and their mean was used for block-level analysis. Scratch loading combines normal indentation and tangential shearing and is therefore suitable for detecting surface instability and early wear responses of enamel.

### 2.6. AFM and SEM Morphological Analyses

AFM was performed using a Dimension Icon atomic force microscope (Bruker, Santa Barbara, CA, USA) in tapping mode. Four separately prepared enamel blocks were examined per group (*n* = 4 block-level replicates). The scanning frequency was 1 Hz, and the working temperature was 25 °C. Three spatially separated representative areas were scanned per block as technical replicates, and their mean was used for block-level analysis. Two-dimensional height maps were used to analyze the equivalent circular diameter distribution of surface aggregates, and Ra, Rq, and Z Range were calculated. AFM was used to detect crystallite-boundary exposure, surface roughening, and nanoscale heterogeneity after deproteinization.

Scratch-damage morphology was examined by SEM using the MIRA LMS microscope. Three representative scratched blocks per group were examined (*n* = 3 per group). After gold sputter coating, secondary electron images of the scratched regions were acquired at a uniform magnification of 2000×. Groove continuity, edge pile-up, crack formation, debris accumulation, and local spallation were compared among groups.

### 2.7. Instrument Calibration and Performance Checks

Before each measurement batch, instrument performance was checked according to the manufacturer’s procedures. TGA mass and temperature signals were verified using the instrument’s reference-check procedure, an FTIR background spectrum was collected before sample scans, and XPS binding energies were referenced to the adventitious C 1s peak at 284.8 eV. For the G200, the Berkovich tip area function and frame compliance were checked using a fused-silica reference standard before the indentation and scratch test series. AFM scanner response was checked using a calibration grating, and thermal drift in creep testing was corrected using the post-unloading hold described in Section 2.4.

### 2.8. Statistical Analysis

Quantitative data are presented as mean ± standard deviation. Statistical analyses were performed using GraphPad Prism 9.0 (GraphPad Software, San Diego, CA, USA) and SPSS 26.0 (IBM Corp., Armonk, NY, USA). Normally distributed data with homogeneous variance were analyzed using one-way analysis of variance followed by Tukey’s post hoc test. Welch’s ANOVA was applied when variance homogeneity was not satisfied. Non-normally distributed data were analyzed using non-parametric tests. A value of *p* < 0.05 was considered statistically significant.

## 3. Results

### 3.1. KOH Treatment Reduced Organic-Related Thermal Mass Loss in Enamel

**Figure 1:**
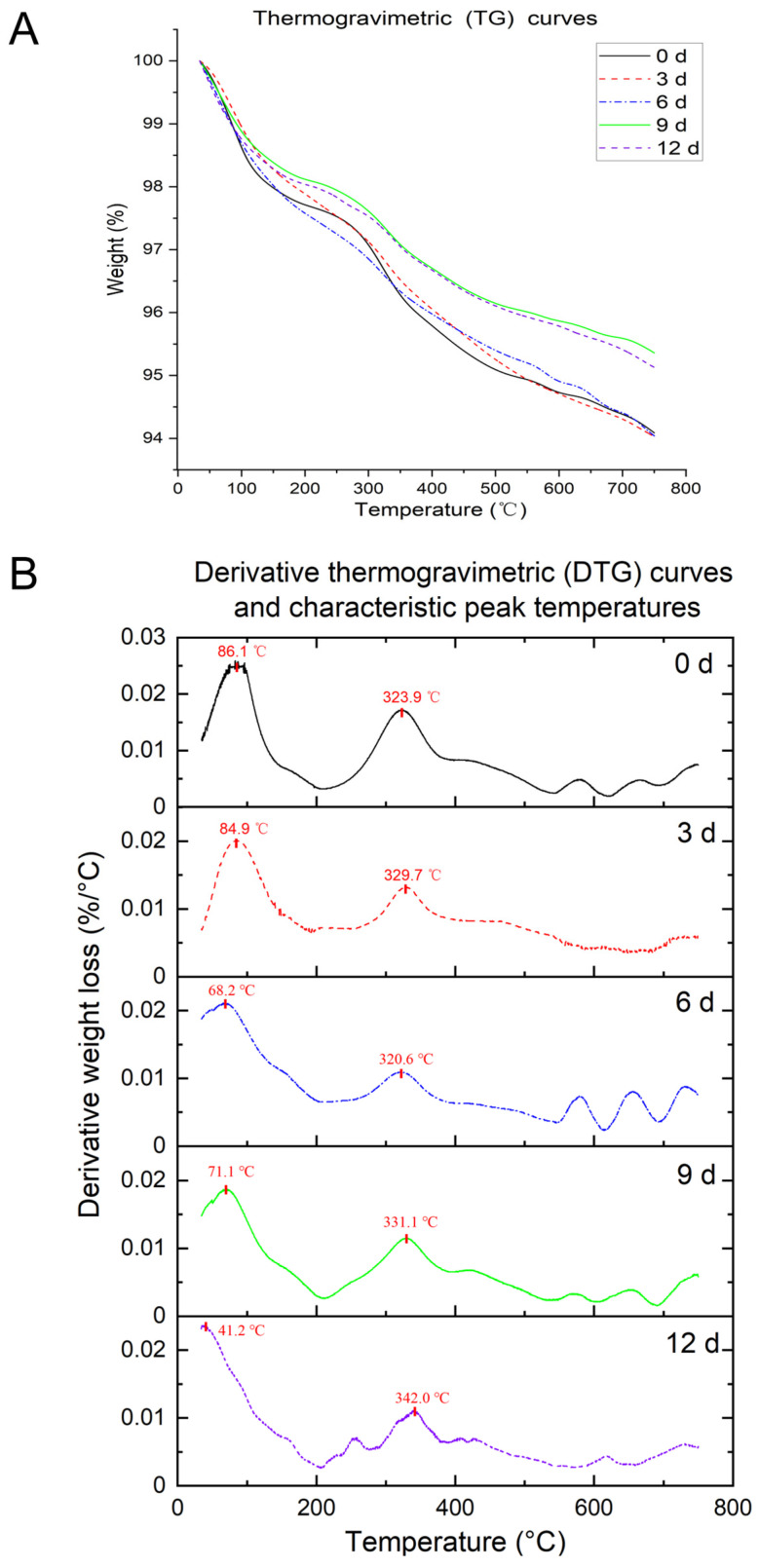
Thermogravimetric validation of the KOH gradient deproteinization model. (**A**) TG curves of mature giant panda enamel after KOH treatment for 0, 3, 6, 9 and 12 days. (**B**) DTG curves showing the main thermal decomposition peaks and the reduced magnitude of the 200–450 °C organic-related mass-loss interval after prolonged KOH exposure.

The TG curves showed continuous mass loss with increasing temperature in all groups, with the major intergroup differences occurring within the 200–450 °C interval, as shown in Figure 1A and Table 1. The mass losses within this interval were 2.32 ± 0.08%, 2.24 ± 0.07%, 1.91 ± 0.06%, 1.74 ± 0.05%, and 1.69 ± 0.09% in the 0-, 3-, 6-, 9-, and 12-day groups, respectively. No significant difference was observed between the 9- and 12-day groups, whereas both groups showed lower mass loss than the 0- and 3-day groups. The final residual mass and the residual mass at 450 °C followed similar trends, indicating that the removal of thermally degradable organic-related components reached a plateau after 9 days of KOH treatment.

The DTG curves showed two major mass-loss peaks in each group, with the second peak located approximately between 320 °C and 342 °C, as shown in Figure 1B. The temperature range of the second peak was comparable among groups, suggesting that deproteinization primarily reduced the magnitude of mass loss in the characteristic interval rather than changing the type of thermal decomposition event. Based on the TG/DTG curves and key parameters, 9 days of KOH treatment was defined as the deproteinization endpoint for subsequent experiments.

### 3.2. FTIR, XPS, and SEM Confirmed Surface Organic-Phase Reduction with Mineral-Framework Preservation

**Figure 2:**
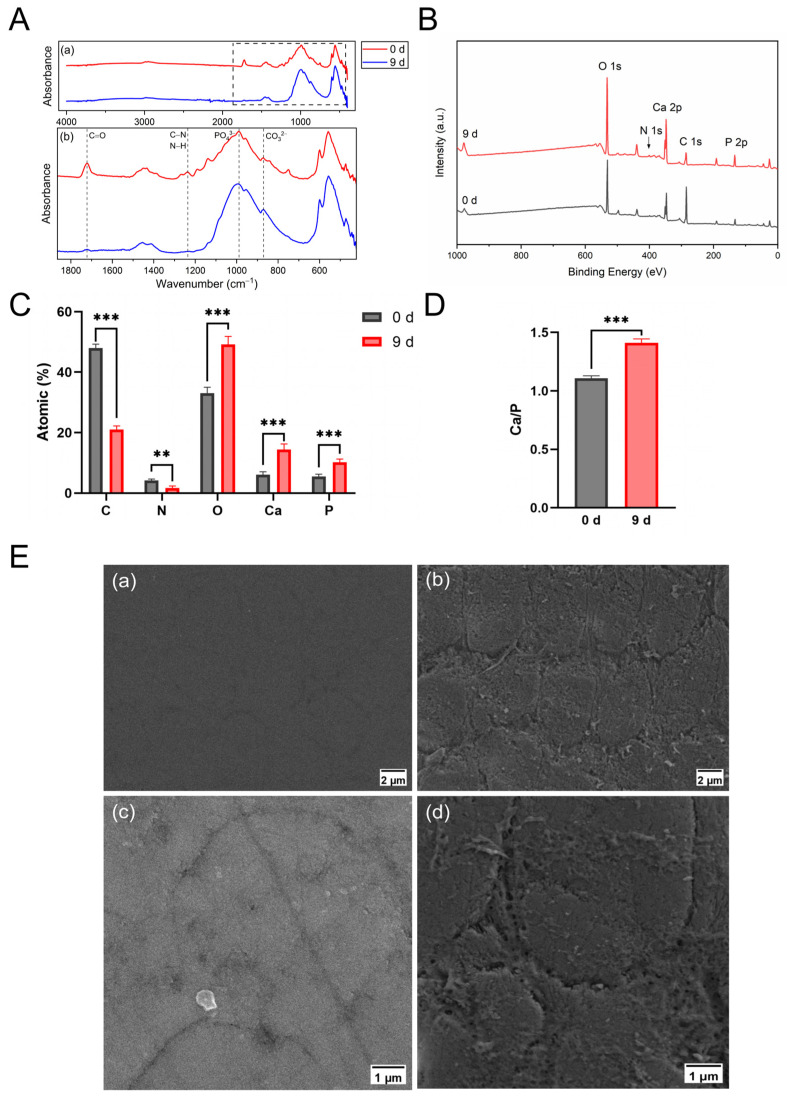
Surface chemical and morphological validation of the 9-day deproteinization endpoint. (**A**) FTIR spectra comparing the 0-day and 9-day groups: (**a**) full spectra and (**b**) the enlarged 1800–500 cm^−1^ region, highlighting organic-related or surface-sensitive bands and retained phosphate/carbonate mineral bands. (**B**–**D**) XPS survey spectra and elemental quantification showing reduced C and N contents and increased mineral exposure after KOH treatment. (**E**) SEM micrographs of the 0-day group at 2000× (**a**) and 10,000× (**c**), and the 9-day group at 2000× (**b**) and 10,000× (**d**), showing surface roughening and interface exposure without gross collapse of the enamel framework. Panels (**C**,**D**): bars show means and error bars show SD (*n* = 3 per group); bracketed asterisks compare the 0-day and 9-day groups: ** *p* < 0.01 and *** *p* < 0.001.

FTIR spectra showed that both the 0-day and 9-day groups retained the main mineral-related absorption bands of enamel, including the phosphate main band near 990 cm^−1^, the carbonate-related band near 872 cm^−1^, and the phosphate ν_4_ doublet near 600 and 557 cm^−1^, as shown in Figure 2A. Compared with the 0-day group, the 9-day group showed reduced absorption at approximately 1722, 1450, 1237, and 1138 cm^−1^, with marked changes at 1237 and 1138 cm^−1^. These results indicate that KOH treatment mainly reduced organic-related or surface-sensitive signals while preserving the major mineral framework of enamel.

XPS survey spectra detected C, N, O, Ca, and P in both groups, as shown in Figure 2B. After 9 days of KOH treatment, the atomic percentages of C and N decreased, whereas those of O, Ca, and P increased. The C content decreased from 47.97% to 21.04%, and the N content decreased from 4.16% to 1.69%. The Ca/P ratio increased from 1.107 to 1.411. These changes support reduced organic coverage and increased exposure of the mineral phase at the enamel surface. SEM images showed that the 0-day group had a relatively flat and continuous surface, whereas the 9-day group displayed increased roughness, clearer interfacial contours, and more micropores and grooves. However, no widespread collapse or extensive structural destruction was observed, as shown in Figure 2E.

### 3.3. Deproteinization Decreased Elastic Modulus and Altered Indentation Creep Response

**Figure 3:**
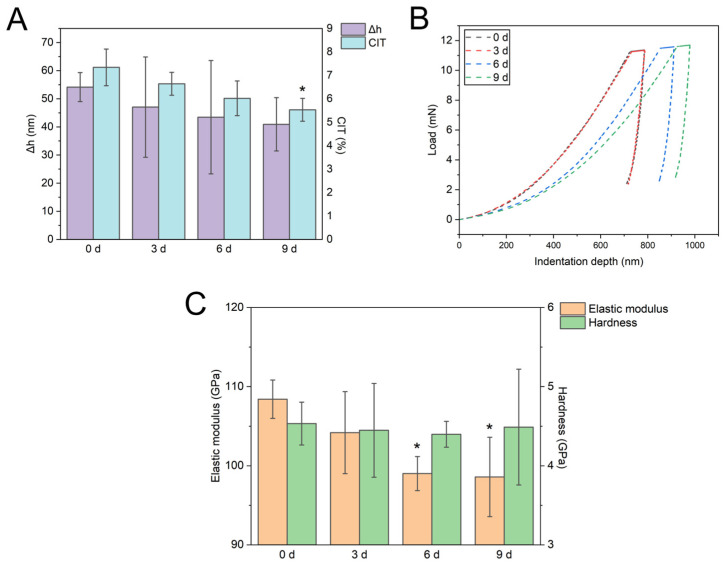
Nanoindentation and indentation creep response of mature giant panda enamel after deproteinization. (**A**) Creep displacement and indentation creep index under constant load. (**B**) Representative load–displacement curves showing increased indentation depth after longer KOH exposure. (**C**) Elastic modulus and hardness values, indicating reduced contact stiffness with relatively preserved hardness. Panels (**A**,**C**): bars show means and error bars show SD (*n* = 4 blocks per group); asterisks compare with the 0-day control: * *p* < 0.05.

Creep displacement (Δh) decreased with increasing deproteinization time, with mean values of 54.15, 47.05, 43.46, and 40.92 nm in the 0-, 3-, 6-, and 9-day groups, respectively; however, the intergroup difference was not statistically significant, as shown in Figure 3A. The indentation creep index was more sensitive to deproteinization, decreasing from 7.34% in the 0-day group to 5.53% in the 9-day group (*p* = 0.009). Load–displacement curves showed that the 6- and 9-day groups reached greater indentation depths under the same loading condition, indicating increased local mechanical compliance after deproteinization, as shown in Figure 3B.

Nanoindentation showed significant differences in elastic modulus among the deproteinization groups (*p* = 0.013), as shown in Figure 3C. Compared with the 0-day group, the 6-day and 9-day groups exhibited significantly lower elastic modulus values. The 3-day group showed an intermediate value but did not differ significantly from the other groups. In contrast, indentation hardness showed only minor changes and no significant intergroup difference. These results indicate that deproteinization primarily affected contact stiffness and elastic response rather than the mineral-dominated resistance to local indentation.

### 3.4. Deproteinization Reduced Scratch-Damage Threshold and Aggravated Permanent Damage

**Figure 4:**
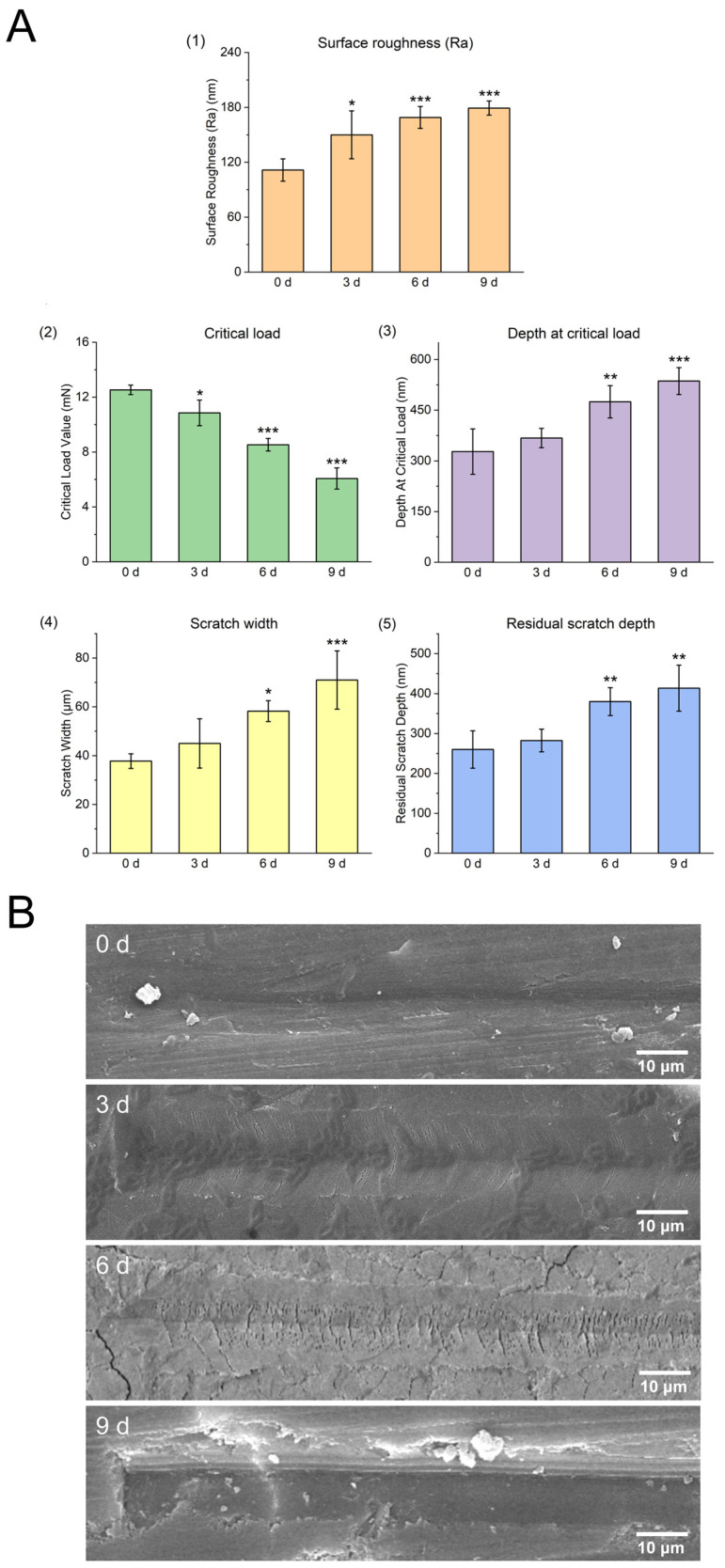
Nanoscratch resistance and scratch-damage morphology of deproteinized giant panda enamel. (**A**) Quantitative nanoscratch parameters: (1) surface roughness, (2) critical load, (3) depth at critical load, (4) scratch width, and (5) residual scratch depth. (**B**) Representative SEM images of scratch tracks showing the transition from shallow plowing grooves to widened grooves, edge instability, debris accumulation and local spallation. Panel (**A**): bars show means and error bars show SD (*n* = 4 blocks per group); asterisks compare with the 0-day control: * *p* < 0.05, ** *p* < 0.01, and *** *p* < 0.001.

Nanoscratch testing revealed a marked effect of deproteinization on surface scratch resistance, as shown in Figure 4A. Surface roughness increased from 111.50 ± 12.18 nm in the 0-day group to 179.25 ± 7.68 nm in the 9-day group. The critical load decreased progressively with treatment duration, from 12.53 ± 0.35 mN in the 0-day group to 6.07 ± 0.77 mN in the 9-day group. The 3-, 6-, and 9-day groups all showed significantly lower critical loads than the 0-day group, indicating that deproteinized enamel entered a damage state under lower normal loading.

The depth at critical load, scratch width, and residual scratch depth increased with deproteinization. The depth at critical load increased from 327.50 ± 67.14 nm to 536.25 ± 39.66 nm; the scratch width increased from 37.75 ± 2.99 μm to 71.00 ± 11.94 μm; and the residual scratch depth increased from 260.00 ± 46.73 nm to 413.75 ± 57.64 nm. These findings demonstrate that deproteinization not only advanced damage initiation but also increased transverse groove expansion and permanent residual damage after unloading, as shown in Figure 4B. In the 0-day group, the main scratch groove was continuous and linear, with a relatively flat groove bottom and limited edge fluctuation. No large defects or extensive spallation was observed, indicating a shallow and regular plowing-type damage mode. In the 3-day group, the groove remained generally continuous, but more transverse or oblique microcrack-like features appeared inside and along the groove edges. The 6-day group showed wider grooves, rougher groove bottoms, reduced edge continuity, and local flake-like or step-like damage. In the 9-day group, the main groove was markedly widened, the boundary became irregular, local edge collapse was observed, and granular or block-like debris accumulated within and around the groove. These features indicate material removal and local brittle failure during scratching. The SEM observations were consistent with the nanoscratch parameters. Increased surface roughness corresponded to reduced baseline flatness and roughened groove edges; decreased critical load corresponded to earlier damage initiation; and increased depth at critical load, scratch width, and residual scratch depth reflected aggravated indentation, transverse expansion, and permanent damage accumulation after unloading.

### 3.5. AFM Revealed Enlarged Surface Aggregates and Increased Three-Dimensional Heterogeneity

**Figure 5:**
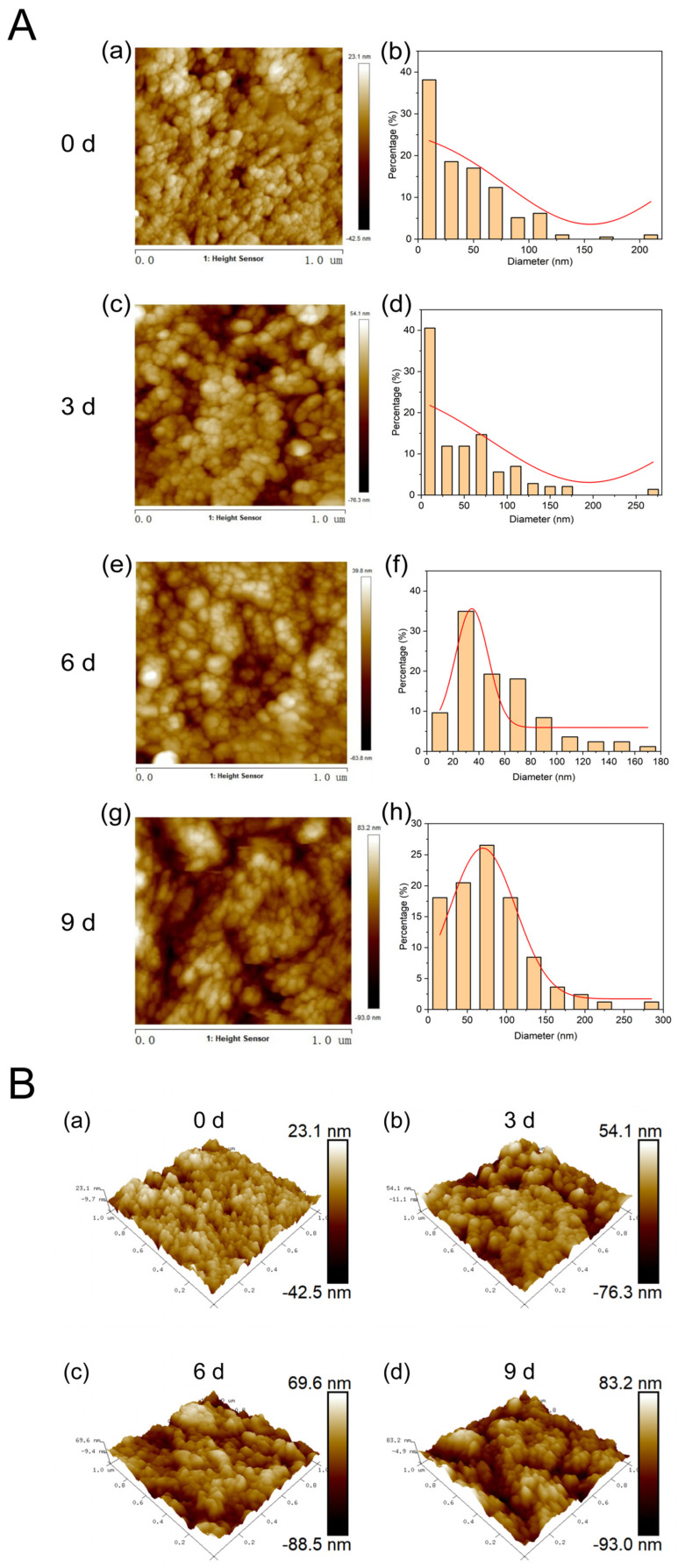
AFM characterization of nanoscale surface roughening after deproteinization. (**A**) Two-dimensional AFM height maps and corresponding equivalent circular diameter distributions of surface aggregates: (**a**,**b**) 0-day, (**c**,**d**) 3-day, (**e**,**f**) 6-day, and (**g**,**h**) 9-day groups. The distributions show a progressive rightward shift with increasing deproteinization time. (**B**) Three-dimensional AFM reconstructions: (**a**) 0-day, (**b**) 3-day, (**c**) 6-day, and (**d**) 9-day groups, showing increased vertical heterogeneity and peak-valley separation.

Two-dimensional AFM images showed that the 0-day group was dominated by small, dense, and relatively uniform aggregate-like protrusions, as shown in Figure 5A. The 3-day group displayed clearer aggregate boundaries and local coalescence. In the 6-day group, high-elevation aggregates became larger and low-elevation gaps formed a more distinct network. In the 9-day group, larger block-like or ridge-like high-elevation structures were observed, and the low-elevation regions deepened and became interconnected. The frequency distribution of equivalent circular diameter shifted progressively to the right with increasing treatment duration. The main peak was located at 0–20 nm in the 0- and 3-day groups, shifted to 20–40 nm in the 6-day group, and further shifted to 60–90 nm in the 9-day group.

Quantitative AFM parameters confirmed increased surface roughness and vertical heterogeneity after deproteinization, as shown in Table 2. In the 0-day group, Ra, Rq, and Z Range were 9.92 ± 1.28, 12.70 ± 1.61, and 100.11 ± 8.13, respectively. In the 9-day group, these values increased to 18.13 ± 0.73, 22.79 ± 0.61, and 185.88 ± 15.91, respectively. Ra and Rq tended to plateau after 6 days, whereas Z Range further increased in the 9-day group, indicating that the vertical extremity difference and three-dimensional complexity continued to increase during late deproteinization.

## 4. Discussion

This study established a KOH-based gradient deproteinization model for mature giant panda enamel and demonstrated that organic-related signals were reduced while the overall mineral framework was retained. The mass loss within 200–450 °C decreased with treatment duration and reached a plateau after 9 days; FTIR showed attenuation of organic-related bands while the phosphate main band and ν_4_ doublet remained clear; XPS showed reduced C and N contents and increased Ca and P contents; and SEM showed interface exposure and surface roughening without gross structural collapse. Together, these findings support 9 days of KOH treatment as an effective deproteinization endpoint. Thermal and spectroscopic analyses of dental hard tissues show that heating and surface chemistry can affect organic-, carbonate-, and phosphate-related signals [19,20]. Elemental and surface-composition studies also indicate that enamel chemistry varies with tissue source and surface treatment [21,22]. Laser and high-temperature studies further show that dental mineral spectra and structure can change when thermal or surface conditions are altered [23,24]. Therefore, the characteristic TG interval should be treated as a composite signal associated with residual proteins, other organic components, structural water, and carbonate-related changes, rather than as an absolute protein mass.

A key mechanical finding was that deproteinization decreased elastic modulus while hardness remained statistically unchanged. Elastic modulus reflects early-stage contact stiffness and elastic response and is sensitive to intercrystalline interface conditions, microstructural continuity, and local hydration/organic-matrix status [2,25]. Hardness is more closely associated with resistance to irreversible indentation beneath the indenter and with the retained mineral framework [3]. Because the mineral framework was largely preserved after KOH treatment, hardness did not decrease to the same extent. The reduced modulus and greater indentation depth are therefore consistent with weakened continuity at intercrystalline or interaggregate interfaces. This pattern suggests that the residual organic phase may contribute to interfacial regulation rather than serving as the principal load-bearing mineral phase [13].

The reduced indentation creep index after deproteinization provides additional insight into the role of the residual organic phase. The lower CIT indicates a reduced capacity for time-dependent deformation accommodation under constant load. In mature enamel, small amounts of organic matter and water may contribute to intercrystalline sliding, local energy dissipation, and microscale deformation coordination [26,27]. Reducing this interfacial phase could limit time-dependent rearrangement under constant load. However, because the present measurements do not directly resolve molecular mobility, the lower creep index should be interpreted as an association with altered interfacial composition rather than as direct proof of a specific energy-dissipation mechanism.

Nanoscratch testing was more sensitive than standard indentation in detecting the functional effects of deproteinization. Scratching combines normal indentation and tangential shearing and can amplify interfacial instability, crack initiation, and edge spallation [28]. The critical load decreased from 12.53 mN to 6.07 mN, indicating that deproteinized enamel entered a clear damage state under a lower normal load. The increased depth at critical load, scratch width, and residual scratch depth further demonstrated earlier damage initiation, greater indentation response at failure, lateral groove expansion, and more severe permanent surface damage. The SEM evidence of increased cracking, groove-edge instability, debris accumulation, and local spallation was consistent with this interpretation. This pattern is compatible with established roles of crack deflection, prism decussation, rod–interrod architecture, and organic-matrix bridging in enamel fracture resistance [29,30]. However, the present data do not isolate the contribution of each mechanism.

AFM provided nanoscale morphological evidence for the mechanical deterioration observed in scratch testing. With increasing deproteinization time, the surface aggregate-size distribution shifted to the right, and Ra, Rq, and Z Range increased. The enamel surface evolved from a fine and dense topography to a heterogeneous surface dominated by larger aggregates and deeper valleys. This change can be interpreted as exposure of intercrystalline boundaries that had been masked or stabilized by organic matter, followed by local aggregation and increased spatial heterogeneity [15,16]. Such roughened surfaces are more likely to generate local stress concentration during scratch contact, thereby promoting crack initiation and edge collapse [31,32].

The residual organic phase of mature enamel is chemically heterogeneous. Plausible sources include enamel-matrix protein fragments and degradation peptides retained in intercrystalline or prism-sheath regions, as well as matrix proteins enriched near the dentin–enamel junction [9,10]. Proteomic analyses of erupted enamel and adjacent matrix layers have identified enamel-derived peptides and other retained proteins [11,12]. Spatial proteomic mapping has shown that enamel-protein composition changes during mineralization and maturation [33]. TGA, FTIR, and XPS cannot identify which specific molecules were removed by KOH, and the present study therefore cannot assign the mechanical changes to a single protein or peptide. Targeted proteomics of sound undecalcified enamel, integrated with spatial sampling, imaging, and mechanical mapping, would be an appropriate next step [34].

This study has limitations. First, the sample size was constrained by the limited availability of dental tissues from an endangered species. Because the six molars originated from only three adults, the findings support the tested specimens but cannot yield population-level estimates or resolve effects of sex, age, tooth position, or individual history; block-level replicates are nested within donors. Larger multi-institutional cohorts and donor-aware analyses are needed to assess generalizability. Second, the tests were performed ex vivo and at local microscale or nanoscale regions, and thus do not fully replicate hydrated cyclic friction and fatigue under natural mastication. The dry, room-temperature measurements standardized the test environment but should not be extrapolated directly to mastication in vivo. Water and protein content can affect enamel creep [27]. Giant panda enamel has also shown hydration-associated self-recovery [8]. Future work should repeat indentation, scratch, and cyclic wear tests in Hank’s balanced salt solution or artificial saliva at approximately 37 °C. Third, although KOH treatment effectively reduced organic-related signals, its potential secondary effects on the surface ionic environment and interfacial hydration cannot be completely excluded. Comparative studies of carnivoran and ursid enamel emphasize that enamel architecture is lineage- and function-dependent [35,36]. Analyses of Hunter–Schreger band enamel further support this relationship [37]. Primate and herbivore studies also show that tooth durability reflects the coupling of geometry, microstructure and local mechanical properties rather than hardness alone [38,39]. Bovine and canine enamel studies are useful comparative models, but their microstructure and wear mechanisms should not be directly extrapolated to giant panda enamel without species-specific validation [40,41]. Future studies should combine wet tribological testing, cyclic fatigue, nano-FTIR, time-of-flight secondary ion mass spectrometry, and finite-element modeling to further correlate organic-phase distribution, interfacial structure, and long-term wear behavior [42,43]. Such work would also help clarify how enamel mechanical properties relate to dietary adaptation across mammalian taxa [44,45]. In addition, the present results cannot establish that reliance on the residual organic phase is unique to giant pandas. A rigorous comparative design should include another ursid as a phylogenetic control and the red panda, cattle, or another species exposed to a high-wear diet as functional comparators. Tooth region, age, storage, hydration, and loading conditions should be matched across species. Recent studies of high-wear beaver enamel and comparative equine dental hard tissues further illustrate the importance of species-specific architecture and oral environment [46,47].

The present SEM and AFM analyses are limited to surface or two-dimensional observations. Rod decussation can redirect enamel cracks [48]. X-ray micro-computed tomography can reveal three-dimensional crack networks that are not apparent at the surface [49]. High-resolution micro-CT before and after controlled loading, ideally combined with in situ loading and image registration, could connect local nanoscale damage to whole-crown architecture, crack pathways, and stress transmission.

## 5. Conclusions

A KOH-based gradient deproteinization model was established for mature giant panda enamel. TGA, FTIR, XPS, and SEM confirmed that 9 days of KOH treatment effectively reduced surface organic-related signals while largely preserving the mineral framework. Based on this model, nanoindentation, creep testing, nanoscratch testing, AFM, and SEM showed that deproteinization decreased elastic modulus and indentation creep index, reduced the scratch critical load, increased permanent scratch damage, and induced enlarged surface aggregates, increased roughness, and more severe crack/spallation features. These findings suggest that the residual organic phase may contribute to surface interfacial continuity, local deformation compatibility, and scratch-damage resistance. Collectively, the residual organic phase may be one material contributor to the adaptation of giant panda enamel to the mechanical demands of a bamboo-based, high-wear diet.

## Figures and Tables

**Table 1:** Main TG/DTG parameters of mature giant panda enamel after KOH treatment.

Group	Terminal Residual Mass (%)	Residual Mass at 200 °C (%)	Residual Mass at 450 °C (%)	Residual Mass Loss Within 200–450 °C Interval (%)
0 d	94.09 ± 0.09	97.71 ± 0.11	95.39 ± 0.10	2.32 ± 0.08 ^a^
3 d	94.03 ± 0.10	97.89 ± 0.09	95.64 ± 0.11	2.24 ± 0.07 ^a^
6 d	94.04 ± 0.08	97.58 ± 0.12	95.67 ± 0.09	1.91 ± 0.06 ^b^
9 d	95.36 ± 0.07	98.12 ± 0.08	96.38 ± 0.07	1.74 ± 0.05 ^c^
12 d	95.13 ± 0.08	98.04 ± 0.10	96.35 ± 0.09	1.69 ± 0.09 ^c^

Values are presented as mean ± SD. Different lowercase letters indicate significant differences within the same column (*p* < 0.05).

**Table 2:** AFM roughness and vertical heterogeneity parameters of mature giant panda enamel after deproteinization.

Group	Ra	Rq	Z Range
0 d	9.92 ± 1.28 ^a^	12.70 ± 1.61 ^a^	100.11 ± 8.13 ^a^
3 d	13.13 ± 1.60 ^b^	16.54 ± 1.93 ^b^	120.73 ± 7.46 ^ab^
6 d	17.33 ± 0.59 ^c^	22.21 ± 0.69 ^c^	160.17 ± 3.89 ^b^
9 d	18.13 ± 0.73 ^c^	22.79 ± 0.61 ^c^	185.8 ± 15.91 ^c^
F value	46.11	52.10	61.03
*p* value	7.32 × 10^−7^	3.73 × 10^−7^	1.55 × 10^−7^

Values are presented as mean ± SD. Different lowercase letters indicate significant differences within the same column (*p* < 0.05).

## Data Availability

The data supporting the findings of this study are included in the article. Further inquiries can be directed to the corresponding author.

## Abbreviations

The following abbreviations are used in this manuscript:

AFM	Atomic force microscopy
CIT	Indentation creep index
DTG	Derivative thermogravimetry
FTIR	Fourier-transform infrared spectroscopy
KOH	Potassium hydroxide
SEM	Scanning electron microscopy
TG	Thermogravimetry
TGA	Thermogravimetric analysis
XPS	X-ray photoelectron spectroscopy

## References

[1] Yilmaz E.D., Schneider G.A., Swain M.V. (2015). Influence of structural hierarchy on the fracture behaviour of tooth enamel. Philos. Trans. R. Soc. A Math. Phys. Eng. Sci..

[2] Habelitz S., Marshall S.J., Marshall G.W., Balooch M. (2001). Mechanical properties of human dental enamel on the nanometre scale. Arch. Oral Biol..

[3] Baldassarri M., Margolis H.C., Beniash E. (2008). Compositional determinants of mechanical properties of enamel. J. Dent. Res..

[4] Bajaj D., Arola D.D. (2009). On the R-curve behavior of human tooth enamel. Biomaterials.

[5] Cuy J.L., Mann A.B., Livi K.J., Teaford M.F., Weihs T.P. (2002). Nanoindentation mapping of the mechanical properties of human molar tooth enamel. Arch. Oral Biol..

[6] Weng Z.Y., Liu Z.Q., Ritchie R.O., Jiao D., Li D.S., Wu H.L., Deng L., Zhang Z. (2016). Giant panda’s tooth enamel: Structure, mechanical behavior and toughening mechanisms under indentation. J. Mech. Behav. Biomed. Mater..

[7] Wu Y., Liu J., Yang Y., Tu S., Liu Z., Wang Y., Peng C., Liu G., Jin Y. (2022). Special architecture and anti-wear strategies for giant panda tooth enamel: Based on wear simulation findings. Front. Vet. Sci..

[8] Liu Z., Weng Z., Zhai Z.F., Huang N., Zhang Z.-J., Tan J., Jiang C., Jiao D., Tan G., Zhang J. (2018). Hydration-induced nano- to micro-scale self-recovery of the tooth enamel of the giant panda. Acta Biomater..

[9] McGuire J.D., Walker M.P., Mousa A., Wang Y., Gorski J.P. (2014). Type VII collagen is enriched in the enamel organic matrix associated with the dentin-enamel junction of mature human teeth. Bone.

[10] Castiblanco G.A., Rutishauser D., Ilag L.L., Martignon S., Castellanos J.E., Mejía W. (2015). Identification of proteins from human permanent erupted enamel. Eur. J. Oral Sci..

[11] Jágr M., Eckhardt A., Pataridis S., Mikšík I. (2019). Proteomic analysis of dentin-enamel junction and adjacent protein-containing enamel matrix layer of healthy human molar teeth. Eur. J. Oral Sci..

[12] Gil-Bona A., Bidlack F.B. (2020). Tooth enamel and its dynamic protein matrix. Int. J. Mol. Sci..

[13] Yahyazadehfar M., Arola D. (2015). The role of organic proteins on the crack growth resistance of human enamel. Acta Biomater..

[14] Lubarsky G.V., D’Sa R.A., Deb S., Meenan B.J., Lemoine P. (2012). The role of enamel proteins in protecting mature human enamel against acidic environments: A double layer force spectroscopy study. Biointerphases.

[15] Koldehoff J., Schneider G.A. (2021). Effect of deproteinization treatments on the structure and mechanical properties of dental enamel. Materialia.

[16] Baumann T., Carvalho T.S., Lussi A. (2015). The effect of enamel proteins on erosion. Sci. Rep..

[17] Taube F., Ylmen R., Shchukarev A., Nietzsche S., Norén J. (2010). Morphological and chemical characterization of tooth enamel exposed to alkaline agents. J. Dent..

[18] Teruel J.D.D., Alcolea A., Hernández A., Ruiz A.J.O. (2015). Comparison of chemical composition of enamel and dentine in human, bovine, porcine and ovine teeth. Arch. Oral Biol..

[19] Xu C., Reed R., Gorski J.P., Wang Y., Walker M.P. (2012). The distribution of carbonate in enamel and its correlation with structure and mechanical properties. J. Mater. Sci..

[20] Kim I.H., Son J.S., Min B.K., Kim Y.K., Kim K.-H., Kwon T.-Y. (2016). A simple, sensitive and non-destructive technique for characterizing bovine dental enamel erosion: Attenuated total reflection Fourier transform infrared spectroscopy. Int. J. Oral Sci..

[21] Viana P.S., Orlandi M.O., Pavarina A.C., Machado A.L., Vergani C.E. (2018). Chemical composition and morphology study of bovine enamel submitted to different sterilization methods. Clin. Oral Investig..

[22] Möhring S., Cieplik F., Hiller K.A., Ebensberger H., Ferstl G., Hermens J., Zaparty M., Witzgall R., Mansfeld U., Buchalla W. (2023). Elemental compositions of enamel or dentin in human and bovine teeth differ from murine teeth. Materials.

[23] Mine A., Yoshida Y., Suzuki K., Nakayama Y., Yatani H., Kuboki T. (2006). Spectroscopic characterization of enamel surfaces irradiated with Er:YAG laser. Dent. Mater. J..

[24] Vargas-Becerril N., García-García R., Reyes-Gasga J. (2018). Structural changes in human teeth after heating up to 1200 °C in argon atmosphere. Mater. Sci. Appl..

[25] Angker L., Swain M.V. (2006). Nanoindentation: Application to dental hard tissue investigations. J. Mater. Res..

[26] He L.H., Swain M.V. (2009). Nanoindentation creep behavior of human enamel. J. Biomed. Mater. Res. Part A.

[27] Koldehoff J., Swain M.V., Schneider G.A. (2023). Influence of water and protein content on the creep behavior in dental enamel. Acta Biomater..

[28] Xia J., Tian Z.R., Hua L., Chen L., Zhou Z., Qian L., Ungar P.S. (2017). Enamel crystallite strength and wear: Nanoscale responses of teeth to chewing loads. J. R. Soc. Interface.

[29] Bajaj D., Arola D. (2009). Role of prism decussation on fatigue crack growth and fracture of human enamel. Acta Biomater..

[30] Yahyazadehfar M., Bajaj D., Arola D.D. (2013). Hidden contributions of the enamel rods on the fracture resistance of human teeth. Acta Biomater..

[31] Bechtle S., Habelitz S., Klocke A., Fett T., Schneider G.A. (2010). The fracture behaviour of dental enamel. Biomaterials.

[32] Lucas P.W., van Casteren A. (2015). The wear and tear of teeth. Med. Princ. Pract..

[33] Green D.R., Schulte F., Lee K.H., Pugach M.K., Hardt M., Bidlack F.B. (2019). Mapping the tooth enamel proteome and amelogenin phosphorylation onto mineralizing porcine tooth crowns. Front. Physiol..

[34] Rexhaj F., Sabel N., Robertson A., Lundgren T. (2023). Evaluation of method parameters for sound undecalcified dental enamel proteomics using liquid chromatography-mass spectrometry. Arch. Oral Biol..

[35] Loch C., Hemm L., Taylor B., Visser I.N., Wiig Ø. (2022). Microstructure, elemental composition and mechanical properties of enamel and dentine in the polar bear Ursus maritimus. Arch. Oral Biol..

[36] Stefen C. (2001). Enamel structure of arctoid Carnivora: Amphicyonidae, Ursidae, Procyonidae, and Mustelidae. J. Mammal..

[37] Lynch C.D., O’Sullivan V.R., Dockery P., McGillycuddy C.T., Rees J.S., Sloan A.J. (2010). Hunter-Schreger Band patterns in human tooth enamel. J. Anat..

[38] Maas M.C., Dumont E.R. (1999). Built to last: The structure, function, and evolution of primate dental enamel. Evol. Anthropol..

[39] O’Brien S., Keown A.J., Constantino P., Xie Z., Bush M.B. (2014). Revealing the structural and mechanical characteristics of ovine teeth. J. Mech. Behav. Biomed. Mater..

[40] Wang C., Fang Y., Zhang L., Su Z., Xu J., Fu B. (2021). Enamel microstructural features of bovine and human incisors: A comparative study. Ann. Anat..

[41] Xiao H., Lei L., Peng J., Yang D., Zeng Q., Zheng J., Zhou Z. (2019). Research of the role of microstructure in the wear mechanism of canine and bovine enamel. J. Mech. Behav. Biomed. Mater..

[42] Yassen G.H., Platt J.A., Hara A.T. (2011). Bovine teeth as substitute for human teeth in dental research: A review of literature. J. Oral Sci..

[43] Schwartz G.T., McGrosky A., Strait D.S. (2020). Fracture mechanics, enamel thickness and the evolution of molar form in hominins. Biol. Lett..

[44] Lucas P.W., Philip S.M., Al-Qeoud D., Al-Draihim N., Saji S., van Casteren A. (2016). Structure and scale of the mechanics of mammalian dental enamel viewed from an evolutionary perspective. Evol. Dev..

[45] Constantino P.J., Lee J.J.W., Gerbig Y., Hartstone-Rose A., Talebi M., Lawn B.R., Lucas P.W. (2012). The role of tooth enamel mechanical properties in primate dietary adaptation. Am. J. Phys. Anthropol..

[46] Hunt T.C., Grejtak T., Kodangal D., Varma S., Rinaldi C.E., Pathak S., Krick B.A., Erickson G.M. (2023). Microstructurally driven self-sharpening mechanism in beaver incisor enamel facilitates their capacity to fell trees. Acta Biomater..

[47] Hertel S., Basche S., Schmidt V., Staszyk C., Hannig C., Sterzenbach T., Hannig M. (2023). Erosion behaviour of human, bovine and equine dental hard tissues. Sci. Rep..

[48] Liu S., Xu Y., An B., Zhang D. (2023). Interaction of rod decussation and crack growth in enamel. Comput. Methods Biomech. Biomed. Eng..

[49] Dumbryte I., Narbutis D., Vailionis A., Juodkazis S., Malinauskas M. (2022). Revelation of microcracks as tooth structural element by X-ray tomography and machine learning. Sci. Rep..

